# *N*-(3-Chloro-2-methyl­phen­yl)succinamic acid

**DOI:** 10.1107/S1600536812022763

**Published:** 2012-05-23

**Authors:** B. Thimme Gowda, Sabine Foro, U. Chaithanya

**Affiliations:** a Department of Chemistry, Mangalore University, Mangalagangotri 574 199, Mangalore, India; b Institute of Materials Science, Darmstadt University of Technology, Petersenstrasse 23, D-64287 Darmstadt, Germany

## Abstract

In the title compound, C_11_H_12_ClNO_3_, the dihedral angle between the benzene ring and the amide group is 44.9 (2)°. In the crystal, mol­ecules form inversion dimers *via* pairs of O—H⋯O hydrogen bonds. These dimers are further linked into sheets parallel to (013) *via* N—H⋯O hydrogen bonds.

## Related literature

For our studies on the effects of substituents on the structures and other aspects of *N*-(ar­yl)-amides, see: Gowda *et al.* (2000 ▶); Chaithanya *et al.* (2012 ▶), of *N*-chloro­aryl­amides, see: Gowda & Rao (1989 ▶); Jyothi & Gowda (2004 ▶) and *N*-bromo­aryl­sulfonamides, see: Gowda & Mahadevappa (1983 ▶), Usha & Gowda (2006 ▶).
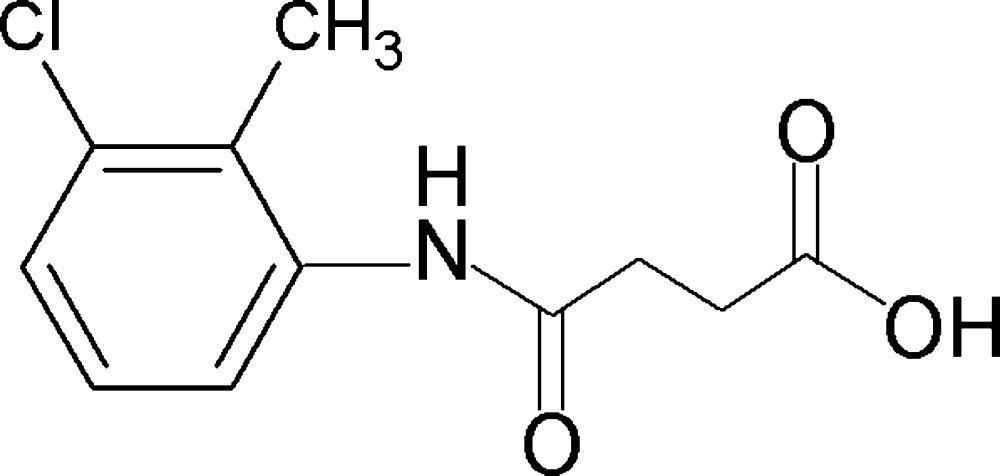


## Experimental

### 

#### Crystal data

C_11_H_12_ClNO_3_
*M* *_r_* = 241.67
Triclinic, 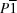

*a* = 4.7672 (9) Å
*b* = 6.297 (1) Å
*c* = 19.135 (3) Å
α = 87.24 (1)°
β = 83.95 (1)°
γ = 88.28 (2)°
*V* = 570.37 (17) Å^3^
*Z* = 2
Mo *K*α radiation
μ = 0.33 mm^−1^
*T* = 293 K
0.40 × 0.20 × 0.02 mm

#### Data collection

Oxford Diffraction Xcalibur diffractometer with a Sapphire CCD detector
Absorption correction: multi-scan (*CrysAlis RED*; Oxford Diffraction, 2009 ▶) *T*_min_ = 0.881, *T*_max_ = 0.994
3270 measured reflections
2072 independent reflections
1578 reflections with *I* > 2σ(*I*)
*R*_int_ = 0.013

#### Refinement

*R*[*F* ^2^ > 2σ(*F* ^2^)] = 0.064
*wR*(*F* ^2^) = 0.127
*S* = 1.20
2072 reflections
152 parameters
2 restraints
H atoms treated by a mixture of independent and constrained refinement
Δρ_max_ = 0.29 e Å^−3^
Δρ_min_ = −0.24 e Å^−3^

### 

Data collection: *CrysAlis CCD* (Oxford Diffraction, 2009 ▶); cell refinement: *CrysAlis RED* (Oxford Diffraction, 2009 ▶); data reduction: *CrysAlis RED*; program(s) used to solve structure: *SHELXS97* (Sheldrick, 2008 ▶); program(s) used to refine structure: *SHELXL97* (Sheldrick, 2008 ▶); molecular graphics: *PLATON* (Spek, 2009 ▶); software used to prepare material for publication: *SHELXL97*.

## Supplementary Material

Crystal structure: contains datablock(s) I, global. DOI: 10.1107/S1600536812022763/bt5924sup1.cif

Structure factors: contains datablock(s) I. DOI: 10.1107/S1600536812022763/bt5924Isup2.hkl

Supplementary material file. DOI: 10.1107/S1600536812022763/bt5924Isup3.cml

Additional supplementary materials:  crystallographic information; 3D view; checkCIF report

## Figures and Tables

**Table 1:** Hydrogen-bond geometry (Å, °)

*D*—H⋯*A*	*D*—H	H⋯*A*	*D*⋯*A*	*D*—H⋯*A*
O3—H3*O*⋯O2^i^	0.83 (2)	1.84 (2)	2.666 (3)	176 (5)
N1—H1*N*⋯O1^ii^	0.83 (2)	2.10 (2)	2.905 (3)	163 (3)

Symmetry codes: (i) 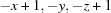; (ii) 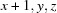.

**Fig. 1.**
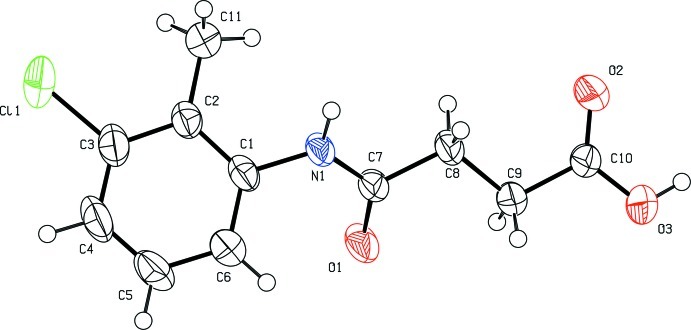
Molecular structure of the title compound, showing the atom labelling scheme. The displacement ellipsoids drawn at the 50% probability level.

**Fig. 2.**
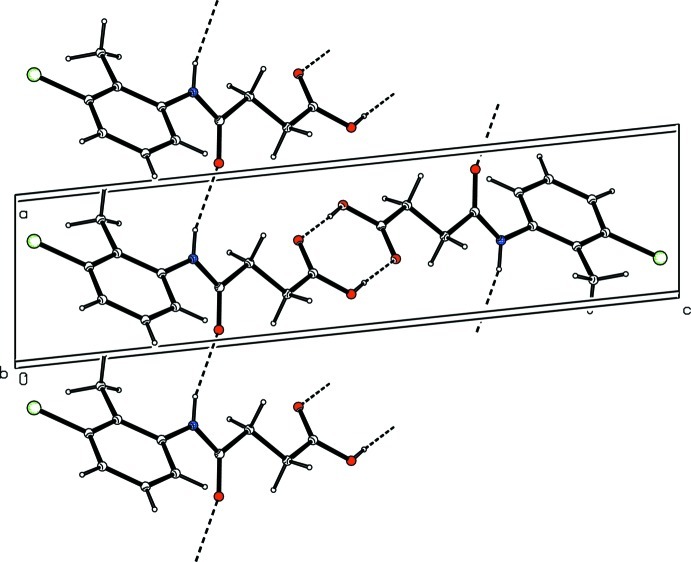
Molecular packing of the title compound with hydrogen bonding shown as dashed lines.

## supplementary crystallographic information

### Comment

As part of our studies on the substituent effects on the structures and other aspects of *N*-(aryl)-amides (Gowda *et al.*, 2000; Chaithanya *et al.*, 2012); *N*-chloroarylsulfonamides (Gowda & Rao, 1989; Jyothi & Gowda, 2004) and *N*-bromoaryl- sulfonamides (Gowda & Mahadevappa, 1983; Usha & Gowda, 2006), in the present work, the crystal structure of *N*-(3-Chloro-2-methylphenyl)succinamic acid has been determined (Fig. 1). The conformation of the N—H bond in the amide segment is *syn* to the *ortho*–methyl and *meta*–Cl in the benzene ring, in contrast to the *anti* conformation observed between the N—H bond and the *meta*–Cl in *N*-(3-chloro-4-methylphenyl)- succinamic acid (I) (Chaithanya *et al.*, 2012).

Further, the conformations of the amide oxygen and the carboxyl oxygen of the acid segments are *anti* to each other and both are *anti* to the H atoms on the adjacent –CH_2_ groups.

The C═O and O—H bonds of the acid groups are in *syn* position to each other, similar to that observed in (I).

The dihedral angle between the phenyl ring and the amide group is 44.9 (2)°, compared to the values of 40.6 (2)° and 44.9 (3)° in the two independent molecules of (I).

In the crystal, the molecules form centrosymmetric dimers via O-H···O hydrogen bonds. These dimers are further linked into sheets parallel to (0 1 3) via intermolecular N–H···O hydrogen bonds. (Table 1, Fig.2).

### Experimental

The solution of succinic anhydride (0.01 mole) in toluene (25 ml) was treated dropwise with the solution of 3-chloro-2-methylaniline (0.01 mole) also in toluene (20 ml) with constant stirring. The resulting mixture was stirred for about one hour and set aside for an additional hour at room temperature for completion of the reaction. The mixture was then treated with dilute hydrochloric acid to remove the unreacted 3-chloro-2-methyl-aniline. The resultant (the title compound) was filtered under suction and washed thoroughly with water to remove the unreacted succinic anhydride and succinic acid. It was recrystallized to constant melting point from ethanol. The purity of the compound was checked and characterized by its infrared spectrum.

Plate like colorless single crystals used in X-ray diffraction studies were grown in ethanolic solution by slow evaporation of the solvent at room temperature.

### Refinement

All H atoms were located in a difference map. The coordinates of the H atoms bonded to N and O were refined with distance restraints of N—H = 0.86 (2) Å and O—H = 0.82 (2) Å, respectively. The other H atoms were positioned with idealized geometry using a riding model with the aromatic C—H = 0.93 Å, methyl C—H = 0.96 Å and methylene C—H = 0.97 Å.

The isotropic displacement parameters of all H atoms were set at 1.2 *U*_eq_(C, N, O) or 1.5 *U*_eq_(C-methyl).

### Crystal data

**Table:**

C_11_H_12_ClNO_3_	*Z* = 2
*M**_r_* = 241.67	*F*(000) = 252
Triclinic, *P*1	*D*_x_ = 1.407 Mg m^−^^3^
Hall symbol: -P 1	Mo *K*α radiation, λ = 0.71073 Å
*a* = 4.7672 (9) Å	Cell parameters from 1394 reflections
*b* = 6.297 (1) Å	θ = 3.2–27.9°
*c* = 19.135 (3) Å	µ = 0.33 mm^−^^1^
α = 87.24 (1)°	*T* = 293 K
β = 83.95 (1)°	Plate, colourless
γ = 88.28 (2)°	0.40 × 0.20 × 0.02 mm
*V* = 570.37 (17) Å^3^	

### Data collection

**Table:**

Oxford Diffraction Xcalibur diffractometer with a Sapphire CCD detector	2072 independent reflections
Radiation source: fine-focus sealed tube	1578 reflections with *I* > 2σ(*I*)
Graphite monochromator	*R*_int_ = 0.013
Rotation method data acquisition using ω and phi scans	θ_max_ = 25.4°, θ_min_ = 3.2°
Absorption correction: multi-scan (*CrysAlis RED*; Oxford Diffraction, 2009)	*h* = −5→5
*T*_min_ = 0.881, *T*_max_ = 0.994	*k* = −7→7
3270 measured reflections	*l* = −22→22

### Refinement

**Table:**

Refinement on *F*^2^	Primary atom site location: structure-invariant direct methods
Least-squares matrix: full	Secondary atom site location: difference Fourier map
*R*[*F*^2^ > 2σ(*F*^2^)] = 0.064	Hydrogen site location: inferred from neighbouring sites
*wR*(*F*^2^) = 0.127	H atoms treated by a mixture of independent and constrained refinement
*S* = 1.20	*w* = 1/[σ^2^(*F*_o_^2^) + (0.0209*P*)^2^ + 0.6764*P*] where *P* = (*F*_o_^2^ + 2*F*_c_^2^)/3
2072 reflections	(Δ/σ)_max_ = 0.004
152 parameters	Δρ_max_ = 0.29 e Å^−^^3^
2 restraints	Δρ_min_ = −0.24 e Å^−^^3^

### Special details

**Table:**

Experimental. Absorption correction: CrysAlis RED (Oxford Diffraction, 2009) Empirical absorption correction using spherical harmonics, implemented in SCALE3 ABSPACK scaling algorithm.
Geometry. All e.s.d.'s (except the e.s.d. in the dihedral angle between two l.s. planes) are estimated using the full covariance matrix. The cell e.s.d.'s are taken into account individually in the estimation of e.s.d.'s in distances, angles and torsion angles; correlations between e.s.d.'s in cell parameters are only used when they are defined by crystal symmetry. An approximate (isotropic) treatment of cell e.s.d.'s is used for estimating e.s.d.'s involving l.s. planes.
Refinement. Refinement of *F*^2^ against ALL reflections. The weighted *R*-factor *wR* and goodness of fit *S* are based on *F*^2^, conventional *R*-factors *R* are based on *F*, with *F* set to zero for negative *F*^2^. The threshold expression of *F*^2^ > σ(*F*^2^) is used only for calculating *R*-factors(gt) *etc*. and is not relevant to the choice of reflections for refinement. *R*-factors based on *F*^2^ are statistically about twice as large as those based on *F*, and *R*- factors based on ALL data will be even larger.

### Fractional atomic coordinates and isotropic or equivalent isotropic displacement parameters (Å^2^)

**Table:**

	*x*	*y*	*z*	*U*_iso_*/*U*_eq_	
Cl1	0.7008 (3)	1.2310 (2)	0.02839 (6)	0.0864 (4)	
O1	0.0716 (4)	0.7206 (4)	0.30679 (13)	0.0603 (7)	
O2	0.5808 (6)	0.1513 (5)	0.42666 (16)	0.0873 (11)	
O3	0.2228 (7)	0.1937 (5)	0.50625 (15)	0.0877 (11)	
H3O	0.286 (10)	0.084 (5)	0.525 (2)	0.105*	
N1	0.5099 (5)	0.8278 (4)	0.26805 (14)	0.0415 (7)	
H1N	0.682 (4)	0.810 (5)	0.2711 (17)	0.050*	
C1	0.4362 (6)	0.9970 (5)	0.22052 (16)	0.0393 (7)	
C2	0.5859 (6)	1.0149 (5)	0.15377 (16)	0.0406 (8)	
C3	0.5154 (7)	1.1899 (6)	0.11106 (18)	0.0514 (9)	
C4	0.3030 (8)	1.3342 (6)	0.1311 (2)	0.0613 (10)	
H4	0.2607	1.4479	0.1009	0.074*	
C5	0.1540 (8)	1.3084 (6)	0.1963 (2)	0.0607 (10)	
H5	0.0081	1.4037	0.2101	0.073*	
C6	0.2212 (7)	1.1408 (5)	0.24147 (18)	0.0492 (9)	
H6	0.1222	1.1245	0.2859	0.059*	
C7	0.3272 (6)	0.7039 (5)	0.30781 (16)	0.0396 (7)	
C8	0.4613 (6)	0.5332 (5)	0.35262 (17)	0.0442 (8)	
H8A	0.5407	0.4215	0.3228	0.053*	
H8B	0.6147	0.5938	0.3739	0.053*	
C9	0.2546 (7)	0.4374 (5)	0.40990 (17)	0.0468 (8)	
H9A	0.1956	0.5453	0.4433	0.056*	
H9B	0.0886	0.3960	0.3891	0.056*	
C10	0.3694 (6)	0.2483 (5)	0.44866 (17)	0.0437 (8)	
C11	0.8049 (7)	0.8513 (6)	0.12881 (19)	0.0561 (10)	
H11A	0.9893	0.9016	0.1341	0.067*	
H11B	0.7911	0.8271	0.0801	0.067*	
H11C	0.7743	0.7208	0.1562	0.067*	

### Atomic displacement parameters (Å^2^)

**Table:**

	*U*^11^	*U*^22^	*U*^33^	*U*^12^	*U*^13^	*U*^23^
Cl1	0.0936 (8)	0.0975 (9)	0.0604 (6)	0.0060 (7)	0.0040 (6)	0.0424 (6)
O1	0.0262 (11)	0.0745 (17)	0.0765 (17)	0.0014 (11)	−0.0078 (11)	0.0376 (14)
O2	0.0694 (18)	0.087 (2)	0.090 (2)	0.0396 (16)	0.0279 (16)	0.0509 (17)
O3	0.095 (2)	0.081 (2)	0.0711 (19)	0.0421 (17)	0.0320 (16)	0.0445 (16)
N1	0.0246 (12)	0.0503 (16)	0.0477 (15)	0.0015 (12)	−0.0063 (12)	0.0195 (13)
C1	0.0326 (16)	0.0368 (17)	0.0493 (19)	−0.0006 (13)	−0.0128 (14)	0.0105 (14)
C2	0.0360 (16)	0.0434 (18)	0.0427 (18)	−0.0029 (14)	−0.0102 (14)	0.0102 (14)
C3	0.051 (2)	0.052 (2)	0.050 (2)	−0.0023 (17)	−0.0102 (16)	0.0185 (17)
C4	0.066 (2)	0.048 (2)	0.069 (3)	0.0076 (19)	−0.019 (2)	0.0244 (19)
C5	0.065 (2)	0.042 (2)	0.075 (3)	0.0200 (18)	−0.014 (2)	0.0042 (19)
C6	0.0474 (19)	0.0468 (19)	0.052 (2)	0.0069 (16)	−0.0061 (16)	0.0051 (16)
C7	0.0284 (16)	0.0479 (19)	0.0415 (17)	0.0030 (13)	−0.0059 (13)	0.0110 (14)
C8	0.0305 (16)	0.0520 (19)	0.0481 (19)	0.0028 (14)	−0.0067 (14)	0.0200 (16)
C9	0.0398 (17)	0.050 (2)	0.0474 (19)	0.0075 (15)	0.0019 (15)	0.0169 (16)
C10	0.0350 (17)	0.0481 (19)	0.0450 (18)	0.0026 (14)	0.0019 (14)	0.0148 (15)
C11	0.050 (2)	0.063 (2)	0.052 (2)	0.0112 (18)	0.0030 (17)	0.0125 (18)

### Geometric parameters (Å, º)

**Table:**

Cl1—C3	1.741 (4)	C4—H4	0.9300
O1—C7	1.222 (3)	C5—C6	1.383 (5)
O2—C10	1.210 (4)	C5—H5	0.9300
O3—C10	1.279 (4)	C6—H6	0.9300
O3—H3O	0.831 (19)	C7—C8	1.512 (4)
N1—C7	1.338 (4)	C8—C9	1.510 (4)
N1—C1	1.427 (4)	C8—H8A	0.9700
N1—H1N	0.834 (18)	C8—H8B	0.9700
C1—C6	1.387 (4)	C9—C10	1.493 (4)
C1—C2	1.397 (4)	C9—H9A	0.9700
C2—C3	1.394 (4)	C9—H9B	0.9700
C2—C11	1.503 (4)	C11—H11A	0.9600
C3—C4	1.376 (5)	C11—H11B	0.9600
C4—C5	1.374 (5)	C11—H11C	0.9600
			
C10—O3—H3O	112 (3)	O1—C7—C8	121.8 (3)
C7—N1—C1	125.5 (2)	N1—C7—C8	114.8 (2)
C7—N1—H1N	119 (2)	C9—C8—C7	112.7 (2)
C1—N1—H1N	115 (2)	C9—C8—H8A	109.0
C6—C1—C2	121.4 (3)	C7—C8—H8A	109.0
C6—C1—N1	119.7 (3)	C9—C8—H8B	109.0
C2—C1—N1	118.8 (3)	C7—C8—H8B	109.0
C3—C2—C1	116.3 (3)	H8A—C8—H8B	107.8
C3—C2—C11	121.9 (3)	C10—C9—C8	114.1 (3)
C1—C2—C11	121.7 (3)	C10—C9—H9A	108.7
C4—C3—C2	122.9 (3)	C8—C9—H9A	108.7
C4—C3—Cl1	117.6 (3)	C10—C9—H9B	108.7
C2—C3—Cl1	119.5 (3)	C8—C9—H9B	108.7
C5—C4—C3	119.3 (3)	H9A—C9—H9B	107.6
C5—C4—H4	120.3	O2—C10—O3	122.5 (3)
C3—C4—H4	120.3	O2—C10—C9	122.8 (3)
C4—C5—C6	120.0 (3)	O3—C10—C9	114.7 (3)
C4—C5—H5	120.0	C2—C11—H11A	109.5
C6—C5—H5	120.0	C2—C11—H11B	109.5
C5—C6—C1	120.0 (3)	H11A—C11—H11B	109.5
C5—C6—H6	120.0	C2—C11—H11C	109.5
C1—C6—H6	120.0	H11A—C11—H11C	109.5
O1—C7—N1	123.3 (3)	H11B—C11—H11C	109.5
			
C7—N1—C1—C6	45.2 (5)	C3—C4—C5—C6	1.0 (6)
C7—N1—C1—C2	−135.5 (3)	C4—C5—C6—C1	−0.9 (6)
C6—C1—C2—C3	2.8 (5)	C2—C1—C6—C5	−1.1 (5)
N1—C1—C2—C3	−176.5 (3)	N1—C1—C6—C5	178.2 (3)
C6—C1—C2—C11	−175.6 (3)	C1—N1—C7—O1	1.3 (6)
N1—C1—C2—C11	5.1 (5)	C1—N1—C7—C8	178.8 (3)
C1—C2—C3—C4	−2.7 (5)	O1—C7—C8—C9	−17.7 (5)
C11—C2—C3—C4	175.7 (4)	N1—C7—C8—C9	164.6 (3)
C1—C2—C3—Cl1	177.4 (2)	C7—C8—C9—C10	171.9 (3)
C11—C2—C3—Cl1	−4.2 (5)	C8—C9—C10—O2	−16.0 (5)
C2—C3—C4—C5	0.8 (6)	C8—C9—C10—O3	165.6 (3)
Cl1—C3—C4—C5	−179.3 (3)		

### Hydrogen-bond geometry (Å, º)

**Table:**

*D*—H···*A*	*D*—H	H···*A*	*D*···*A*	*D*—H···*A*
O3—H3*O*···O2^i^	0.83 (2)	1.84 (2)	2.666 (3)	176 (5)
N1—H1*N*···O1^ii^	0.83 (2)	2.10 (2)	2.905 (3)	163 (3)

Symmetry codes: (i) −*x*+1, −*y*, −*z*+1; (ii) *x*+1, *y*, *z*.
